# Safety and Ethics in Endoscopic Studies in Children: Evidence From the BEECH Study in Zambia

**DOI:** 10.1093/tropej/fmaa074

**Published:** 2020-11-14

**Authors:** Kanta Chandwe, Beatrice Amadi, Miyoba Chipunza, Masuzyo Zyambo, Paul Kelly

**Affiliations:** 1 Tropical Gastroenterology & Nutrition group, University of Zambia School of Medicine, 10101 Lusaka, Zambia; 2 Department of Anaesthesia, University of Zambia School of Medicine, 10101 Lusaka, Zambia; 3 Barts & The London School of Medicine, Queen Mary University of London, London E1 2AT, UK

## Abstract

**Background:**

Environmental enteropathy is an example of a poorly-understood intestinal disorder affecting millions of children worldwide, characterized by malabsorption and stunting. Although there is increasing interest in non-invasive means of assessing intestinal structure and function, the potential value of intestinal biopsy for histology, immunostaining, RNA sequencing and epigenetic work means that endoscopic biopsy remains extremely valuable. We here report our experience in the BEECH (Biomarkers of Environmental Enteropathy in CHildren) study of stunting in Zambia, in the belief that it may help address the knowledge gap regarding the safety of endoscopic biopsy in vulnerable young children.

**Methods:**

We report our experience of safety in 119 children undergoing endoscopic biopsy in the BEECH study in Lusaka Children’s Hospital, Lusaka, and discuss ethical considerations in this light.

**Results:**

Upper gastrointestinal endoscopy was performed on children with stunting (length-for-age z score -2 or less) not responsive to nutritional interventions. Conscious sedation was provided by anaesthetists. Of 119 children, 5 (4%) developed transient desaturation, but no serious adverse events were experienced; no clinical, demographic or anaesthetic characteristics were identified as predictive of desaturation. Two children derived clinically useful information from the endoscopy, one life-saving. Of 105 lactase tests, 59 (54%) showed hypolactasia.

**Discussion:**

Children with stunting underwent endoscopy safely, and some derived clinical benefit. Safety and the possibility of clinical benefit are usually felt to be preconditions for the ethical justification for endoscopy for research in children, and we believe that these conditions were met in this study.

## INTRODUCTION

Environmental enteropathy (EE) is an asymptomatic disorder of the small intestine which is currently believed to underlie three severe clinical problems of major global health significance [1–3]. The first, stunting, is estimated to affect at least 150 million children worldwide [4] and 37% of infants in Africa [5]. It is due to long-term nutritional inadequacies, but responds only very partially to nutritional supplementation [6–9]. This counter-intuitive finding, confirmed in several continents [6–9], suggests that there may be a rate-limiting biological constraint on nutrient utilization for growth, and there is great interest in determining if EE is that constraining factor [10]. Second, micronutrient deficiencies are widespread [4], and it is likely that EE contributes to these deficiencies, especially when intakes are only marginally sufficient. Third, there is a clear geographical association between EE and failure of responses to oral vaccines [11]. Although little direct evidence exists to incriminate EE as the principal cause of vaccine failure, there is indirect evidence to support this idea [12, 13]. Interactions between viral, bacterial and protozoal pathogens in determining enteropathy and nutrition are complex [14, 15], and probably underlie at least some of the impairment in oral vaccine responses [16].

There is great interest in evaluating EE, but it is difficult to measure. Histological assessment has historically been focussed on villus: crypt ratio, either alone or as part of a comprehensive morphometric measurement of villi and crypts, and/or an assessment of mucosal inflammation. A review of the literature found five studies in which villus height measurements were made in children; three of these were conducted in Africa [17–19], one in the UK [20] and one in both [21] (Table 1). The paucity of data from only five countries reflects the reluctance of the research community to undertake endoscopic mucosal biopsy from children.

**Table:** **Table 1:** *Previous studies of mucosal morphometry in children*

First author	Country	Nutrition	*n*	VH (μm)	CD (μm)	VH: CD ratio	References
Cook	Uganda	Normal^a^	20	321 (271–359)^b^	–	–	[17]
Gendrel	Gabon	SAM	13	218 (43)	154 (17)	–	[18]
Amadi	Zambia	SAM	22	200 (57)	164 (30)	1.25 (0.39)	[19]
Penna	UK	Normal	24	332 (45)	169 (28)	2.0 (0.35)	[20]
Campbell	Gambia	SAM	38	243 (68)	278 (69)	0.82 (0.25)	[21]
	UK	Normal	19	355 (35)	170 (20)	2.1 (0.3)	

a Normal nutritional status but had experienced severe malnutrition 4 years previously. Values shown are mean (SD) except where marked.

b Median (range).

For this reason, the majority of studies of enteropathy from different geographical locations have employed one of the various available tests based on lactulose permeation as a measure of mucosal damage [22–24]. However, current technology for tissue analysis allows much greater insights into mucosal biology than has hitherto been possible, with sophisticated multi-colour immunostaining [25], flow cytometry [26] and transcriptomic analysis [27] all now possible on mucosal biopsies. The potential insights into pathophysiology from endoscopic biopsy are considerable. We set up the BEECH study, including mucosal biopsies from children with stunting, in the hope that we would be able to bring fresh technology to bear on EE, while at the same time deriving clinical benefit for some of the children by finding specific disorders which could be amenable to therapy. Here, we report the safety of the first 119 procedures, and reflect on the ethical stance we took when designing the study.

## BEECH STUDY DESIGN

This study builds on previous experience of endoscopy in severely malnourished children [19, 28]. In the BEECH study, children were recruited between 0 and 18 months of age in communities in south Lusaka where stunting is common, and from which historically a large proportion of malnourished children admitted to the central paediatrics hospital have been drawn. This part of the city has benefitted from a major programme designed to identify Moderate Acute Malnutrition (MAM) and Severe Acute Malnutrition (SAM) early [29], and BEECH is adding to that the capacity for screening from the earliest weeks of life. The aim was to identify children in their homes who are <1 SD for weight for age (WAZ score < −1), assess them thoroughly for stunting [length-for-age z score (LAZ) −2 or less], wasting [weight-for-length z score (WLZ) −2 or less] or oedematous malnutrition, provide nutritional rehabilitation in the form of a daily ration of high energy protein supplement (corn–soy blend), an egg and a micronutrient sprinkle (Nutrimix, Hexagon Nutrition, Mumbai). Children who continued to have LAZ and/or WLZ ≤ −2 SD after 3–6 months of nutritional supplementation were classified as non-responders and further investigations were instituted, including an upper gastrointestinal (GI) endoscopy.

Children identified with growth failure not responding to nutritional supplementation were admitted to hospital the day prior to endoscopy for clinical examination, full blood count and International Normalised Ratio (INR) , to exclude children likely to have increasing bleeding risk. These results were reviewed by two of the investigators so that children at increased risk of bleeding could be investigated and treated. On the morning of endoscopy, they were evaluated by an anaesthetist and fasted for 3 h prior to the procedure. Anaesthesia was intended to provide deep sedation.

## SAFETY

Between September 2016 and December 2018 we prepared 122 children for endoscopy, but endoscopy was deferred in children with Hb <9 g/dl (*n* = 1), elevated leucocyte count (*n* = 1) or INR >1.3 (*n* = 1), and endoscopy was successfully performed on 119 children (58 boys, 61 girls, mean age 12.4 months). Of these children, 118 had non-responsive stunting (length-for-age z score ≤-2) and 8 children had wasting non-responsive wasting (weight-for-length z score ≤−2); 7 had both. Median (IQR) LAZ scores were −3.3 (−3.9, −2.8), with range −5.0 to −1.7. The child who did not have stunting had a weight-for-length z score of −3.1.

One endoscopist performed all of the endoscopy procedures. Anaesthesia for these endoscopic procedures was provided by a total of 14 anaesthetists; the number of procedures by each doctor varied from 1 to 20. Ketamine was used in 80 of 104 procedures for which records are complete, with a median (IQR) total dose of 18.5 (10, 25) mg. Propofol was used in 34 procedures, with a median (IQR) dose of 15 (15, 20) mg. Midazolam was used in 26 procedures, with a median (IQR) dose of 0.7 (0.5, 1.0) mg. A combination of ketamine and midazolam was used in 13 children, and midazolam with propofol also in 13 children; a ketamine–propofol combination was used in 17.

No serious adverse events occurred. A total of five episodes of desaturation were experienced, all of them transient (Table 2). In four of five instances the procedure was discontinued to permit high-flow oxygen therapy and monitoring, and then resumed once saturation improved. No other adverse events occurred. The episodes of desaturations appeared to be idiosyncratic as no clinical or anaesthetic characteristics were found which correlated with desaturations.

**Table:** Table 2: *Clinical characteristics and details of sedation in five children with transient desaturation during endoscopy*

ID	Sex	Age (m)	LAZ	WLZ	HIV	Hb (g/l)	Dose (mg)			Saturation event			Interrupted?	Time to recovery (s)	Anaesthetist
							M	K	P	SpO_2_ before procedure (%)	Time to desaturation (s)	SpO_2_ nadir (%)			
31	F	14	−3.38	−2.36	N	124	1	10	0		120	66	Yes	<120	A
80	M	17	−3.09	−0.53	N	104	1	18	0	98	60	45	No	<180	B
141	F	20	−4.45	−0.95	P	117	0.5	10	10	96	120	48	Yes	<120	C
224	M	10	−3.38	1.80	N	108	0	10	10	99	45	50	Yes	<360	D
298	F	14	−2.70	−1.42	N	111	0	35	0	99	120	51	Yes	<120	B

LAZ, length-for-age z score; WLZ, weight-for-length z score; M, midazolam; K, ketamine; P, propofol; SpO2, peripheral oxygen saturation (%).

Clinical benefit directly from the endoscopic procedure was obtained in only two children, one with ascariasis and one who was found to have oesophageal candidiasis. The latter child was born of an HIV seropositive mother but found not to have circulating HIV RNA by PCR at 6 weeks of age and was offered no further follow-up. Prompted by the finding of oesophageal candidiasis, an AIDS-defining opportunistic infection, these tests were repeated and the child found to have active HIV infection. The child is now doing well on anti-retroviral therapy. In addition, 105 children had lactase tests performed, and of these 59 (54%) showed mild or severe hypolactasia.

## ETHICAL CONSIDERATIONS

The BEECH protocol was approved by the University of Zambia Biomedical Research Ethics Committee (ref 006-02-16) on 31 May 2016. All investigators have deliberated on whether, and under what circumstances, endoscopic procedures for research purposes are justifiable in children with malnutrition. As always the principles of ethical medical research dictate that the rights and safety of the individual cannot be overruled by the desire to establish a body of knowledge which will benefit others.

The consensus among the authors of this article is that endoscopic procedures in children for research can only be justified if the individual child has a reasonable probability of benefitting from the information obtained. In the case of a malnourished child with diarrhoea, or non-response to standard nutritional rehabilitation, endoscopic examination may reveal conditions such as oesophageal candidiasis or stricturing, and the biopsy itself may enable a diagnosis of an intestinal infection (e.g. giardiasis or cryptosporidiosis) which may elude detection by routine stool microscopy. It is well known that for many pathogens, such as *Giardia* or *Cryptosporidium* spp., stool microscopy is insensitive [30]. Coeliac disease is also best diagnosed with endoscopic biopsy, though there is a body of opinion that serological diagnosis may sometimes be sufficient if unequivocal and consistent with the clinical picture [31]. For standard indications, including failure to thrive, reflected in current NASPGHAN and ASGE guidelines (ASGE) and elsewhere [32] upper GI endoscopy has a high diagnostic yield (79%) and makes a significant contribution to management [33, 34]. It is also the only definitive way that certain disorders can be diagnosed, including food allergy [35], sucrase-isomaltase deficiency [36] or tufting or other forms of enteropathy [25]. The frequency with which these may contribute to failure to thrive in low- and middle-income countries, although probably low, is unknown. We found two intestinal disorders which had not been made by other means. We also found mild or severe lactase insufficiency in 54% of children; while this is helpful in determining appropriate feeds for the child it would have made only a modest difference to clinical outcome. It should be emphasized that stunting is not a benign disorder, associated as it is with increased mortality [37, 38]. Endoscopy findings and the results of biopsies must be made available in a timely way so that appropriate treatment may be provided. The high prevalence of hypolactasia may suggest that avoidance of milk-based feeds may be helpful. This may have policy implications, as standard feeds for treatment of SAM in hospitalized children (F75 and F100) contain lactose. Children in the BEECH study with lactose intolerance were switched onto commercial lactose-free feeds but these are expensive and not sustainable. Further work is needed on this point.

Endoscopy is safe when performed by experienced operators in well-equipped units [33, 34], but is never without risk. The major risk is related to the sedation required for a good endoscopic examination, and there are guidelines to help in delivering safe sedation with benzodiazepines, opiates, ketamine or propofol [39–41] so that this risk is now low. Generally, sedation for endoscopic procedures does not include tracheal intubation, but to our knowledge the risk/benefit analysis of leaving the airway unprotected has not been quantified. Other risks, which have not been robustly quantified in children undergoing diagnostic upper GI endoscopy, include haemorrhage, perforation or duodenal haematoma [42]. The risk/benefit balance we have adopted is consistent with another study of EE in Bangladesh [43].

We readily acknowledge the limited power of this study to demonstrate safety (i.e. absence of serious adverse outcomes). There exists a statistical tool which is helpful in estimating the likelihood of serious adverse events when a series of procedures reveals no serious adverse events: the ‘rule of three’ [44, 45]. This states that the upper confidence limit of the range of probabilities of a serious adverse event is 3/*n*, where *n* is the number of event-free procedures which has been performed. Importantly, this only applies when the numerator is zero, and when *n *>* *50 [46]. Applying the ‘rule of three’ to our data suggest that the real frequency of serious adverse events is likely to be <3%. Given that the frequency of mild, transient desaturations was 4%, is this safe enough to permit procedures which are likely to confer only limited benefit to the individual child? We believe so, as one child gained benefit from the procedure, over 50% obtained useful clinical information, and no child suffered significant harm. Nevertheless, the balancing of risks and benefits when investigating vulnerable children requires constant vigilance, thorough preparation prior to the procedure and careful monitoring of outcomes.

Having observed differing preferences in the choices and doses of anaesthetic drugs at our centre, we have established a working group to set up a standardized sedation protocol for endoscopy in our unit. We also recommend and support efforts to find sustainable lactose-free feeds that may benefit some children undergoing nutritional rehabilitation in underprivileged populations.
